# Comprehensive Spatial Profile of the Orphan G Protein Coupled Receptor GPRC5B Expression in Mouse Brain

**DOI:** 10.3389/fnins.2022.891544

**Published:** 2022-06-23

**Authors:** Wenqi Fu, Luca Franchini, Cesare Orlandi

**Affiliations:** Department of Pharmacology and Physiology, University of Rochester Medical Center, Rochester, NY, United States

**Keywords:** G protein coupled receptor (GPCR), orphan GPCR, GPRC5B, RNAscope assay, neuropharmacology

## Abstract

Orphan G Protein Coupled Receptors (GPCRs) are GPCRs whose endogenous ligands are unknown or still debated. Due to the lack of pharmacological modulators, the physiological function of orphan GPCRs is understudied. However, relevant physiological roles associated with orphan GPCRs have been revealed by analysis of animal models and genome wide association studies illuminating an untapped potential for drug discovery. G Protein Coupled Receptor class C Group 5 Member B (GPRC5B) is among the most expressed GPCRs in the central nervous system. Thus, the expression profiling of GPRC5B is an essential step toward understanding GPRC5B function in health and disease. In this study, we generated new GPRC5B polyclonal antibodies and investigated the expression levels of GPRC5B across different organs and brain regions. We identified high levels of GPRC5B glycosylation both in transfected cells and in mouse brain. Moreover, *in situ* hybridization imaging analysis indicated that *Gprc5b* was expressed at the highest level in olfactory bulb, hippocampus, cerebellum, and pons. To dissect expression within various neuronal populations, we conducted a comprehensive spatial profiling of *Gprc5b* across excitatory and inhibitory neuronal types in medial prefrontal cortex, motor cortex, hippocampal regions, hypothalamus, and cerebellum. Overall, we discovered that GABAergic neurons displayed higher *Gprc5b* expression levels than glutamatergic neurons in most of the analyzed regions with the important exception of the hippocampal dentate gyrus. Overall, the expression analysis of GPRC5B in mouse brain will guide functional studies ultimately positioning GPRC5B in pathophysiological mechanisms and drug discovery.

## Introduction

**FIGURE 1:**
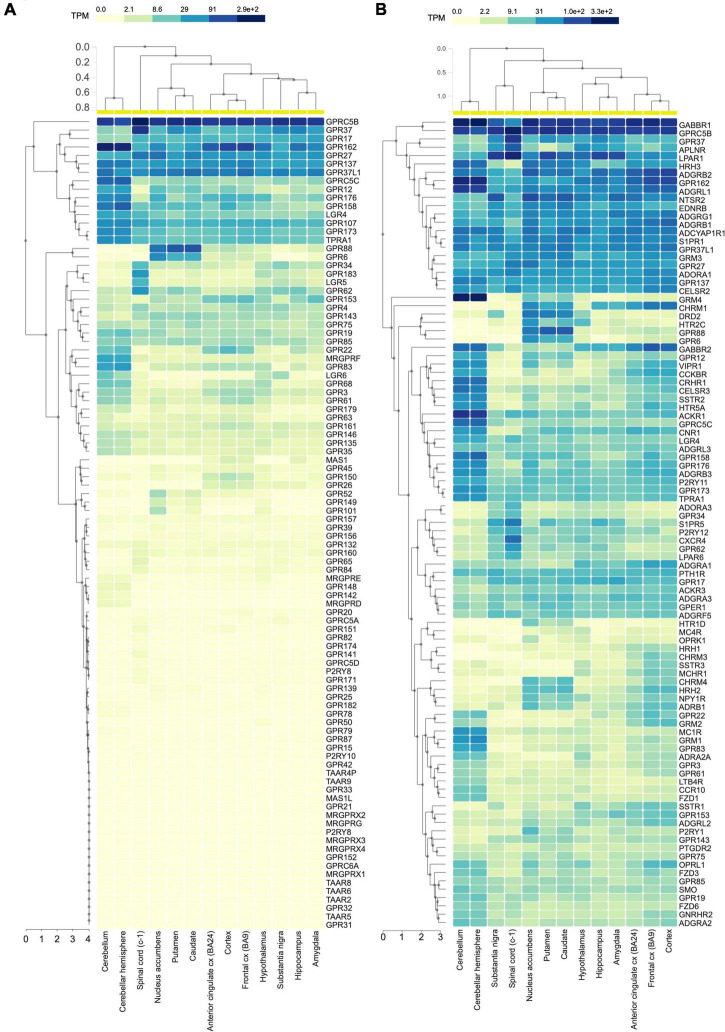
GPRC5B is the most expressed oGPCR in the CNS. **(A)** Human RNA sequencing data analyzing 96 orphan GPCRs from the GTEx portal show high expression levels of GPRC5B. **(B)** GTEx portal RNA sequencing data reporting expression levels across brain regions of the 100 most expressed GPCRs in the CNS.

In mammals, G protein coupled receptors (GPCRs) constitute the largest family of transmembrane proteins, with many members being highly expressed or enriched in the central nervous system (CNS). GPCR members of the class C include metabotropic glutamate receptors, GABAB receptors, calcium sensing receptors, type 1 taste receptors, and a group of GPCRs whose endogenous ligands have not been characterized yet and are therefore dubbed as orphans (oGPCRs). Among these class C oGPCRs, a group of four receptors, GPRC5A, GPRC5B, GPRC5C, and GPRC5D, were originally identified as retinoic acid inducible GPCRs in different cell lines including head and neck squamous cancer cells (Cheng and Lotan, 1998; Bräuner-Osborne and Krogsgaard-Larsen, 2000; Robbins et al., 2000; Bräuner-Osborne et al., 2001). Unlike other class C GPCRs, GPRC5 members bear a short extracellular N-terminus, therefore they lack the Venus flytrap ligand binding pocket typically found in class C GPCRs (Bräuner-Osborne and Krogsgaard-Larsen, 2000). Patterns of tissue distribution are distinct among the four GPRC5 family members with GPRC5B and GPRC5C being enriched in the CNS (Cheng and Lotan, 1998; Robbins et al., 2000, 2002; Xu et al., 2005; Imanishi et al., 2007), whereas GPRC5A is expressed abundantly and specifically in the lung (Cheng and Lotan, 1998; Robbins et al., 2000; Xu et al., 2005), and GPRC5D in skin, pancreas, and upregulated in myeloma (Bräuner-Osborne et al., 2001; Inoue et al., 2004; Atamaniuk et al., 2012). In the CNS, human RNA sequencing data show that GPRC5B is the most expressed among all the oGPCRs (Figure 1A). Strikingly, exploring the mRNA abundance of the 100 most expressed GPCRs in the CNS, GPRC5B is still at the top of the list with expression levels comparable to GABAB1 receptor (Figure 1B).

Abundance in the CNS suggests a potential functional role of GPRC5B in the control of neurophysiological processes. Studies carried out in mice where expression of GPRC5B was genetically ablated revealed a partial neonatal lethality resulting in almost 50% mortality of GPRC5B knockout mice before 4 weeks of age (Sano et al., 2011). The survived mice showed a diminished spontaneous activity before light onset and decreased overall activity in the open field test compared to wild type littermates (Sano et al., 2011). Moreover, an increased time spent in the center of the open field arena suggested an anxiolytic phenotype that has never been confirmed with additional behavioral studies (Sano et al., 2011). Further studies using conditional ablation of GPRC5B in the cerebellar Purkinje cells identified deficits in long-term motor learning *via* dysfunction of synaptic plasticity, as well as motor coordination problems due to the disruption of development of Purkinje cell distal axons and synaptic formation with cerebellar nuclear neurons (Sano et al., 2018). Furthermore, transcriptional analysis in post-mortem brain from patients suggested an involvement of GPRC5B in signaling pathways leading to bipolar disorders (BPD) and major depressive disorders (MDD) (Aston et al., 2005; Tomita et al., 2013). Finally, genome-wide association studies (GWAS) sorted out that intronic regions of GPRC5B may be involved in the etiology of attention deficit hyperactivity disorder (ADHD) (Albayrak et al., 2013). Altogether, these evidence point to a relevant physiological role for GPRC5B in the CNS that demands further investigations. Physiological roles of GPRC5B have also been examined in peripheral systems. A thorough study using GPRC5B smooth muscle cell conditional knockout mice, revealed a critical role in regulating contractility and differentiation of cardiomyocytes, mechanistically associated with inhibiting prostacyclin signaling (Carvalho et al., 2020). Another study indicated that GPRC5B may be a major node in adipose signaling systems linking diet-induced obesity to the type 2 diabetes (Kim et al., 2012). Lastly, GPRC5B was found involved in the modulation of inflammatory responses in the glomerulus *via* NF-κB pathway (Zambrano et al., 2019). An increasing amount of data supporting a plethora of functions for GPRC5B throughout different systems makes investigations related to GPRC5B in the CNS, where it is enriched, even more significant.

Here, we performed a comprehensive profiling of *Gprc5b* expression in eleven mouse brain regions as well as an in-depth study of its distribution within distinct neuronal populations. At the protein level, we generated and validated a new anti-GPRC5B polyclonal antibody which facilitated the expression characterization across tissues and various brain regions. Ultimately, at the subcellular level, a biochemical fractionation assay provided evidence for localization of GPRC5B both in intracellular compartments and at the post-synaptic density (PSD). Overall, the present study produced a comprehensive expression profile of GPRC5B in mouse brain and in neuronal subpopulations, which improved both the breadth and depth of knowledge about GPRC5B and will facilitate future functional studies.

## Materials and Methods

### Animals

C57BL/6J mice were housed in a temperature and humidity-controlled room at the vivarium in the University of Rochester on a 12:12-h light/dark cycle (lights off at 18:00 h) provided with food and water *ad libitum*. Male and female adult mice were used for all the experiments. All procedures were pre-approved and carried out in accordance with the University Committee on Animal Resources (UCAR) at the University of Rochester.

### RNA Sequencing Data Sources

The human RNA sequencing data used for the analyses described in this manuscript were obtained from the Genotype-Tissue Expression (GTEx) portal on February 19, 2022. The GTEx Project was supported by the Common Fund of the Office of the Director of the National Institutes of Health, and by NCI, NHGRI, NHLBI, NIDA, NIMH, and NINDS.

### GPRC5B Antibody Generation

Polyclonal antibodies against GPCR5B (GPRC5B-CT) were affinity-purified from rabbit sera after immunization with synthetic peptides (Pocono Rabbit Farm & Laboratory, Inc., Canadensis, PA, United States). Briefly, 4 mg of a synthetic peptide from a conserved amino acid strand within the C terminus sequence of GPRC5B, identical in human, mouse, and rat (C-PNYFDTSQPRMRETAF) were generated by Pocono Rabbit Farm & Laboratory and covalently immobilized using UltraLink immobilization kit (Thermo Scientific, Waltham, MA, United States). Antibodies against GPRC5B were then purified by affinity chromatography from immune sera according to the following protocol: 20 ml of rabbit serum were diluted in 250 ml of PBS and filtered through a 0.22 μm vacuum filter. The filtered serum was then run through the prepared affinity column at a speed of ∼1 ml/min for 2 days using a peristaltic pump at 4°C. The column was then washed with 50 ml of 500 mM NaCl in PBS, followed by one wash with 100 ml of PBS. Antibodies were subsequently eluted from the column using 100 mM NaH_2_PO_4_ (pH 2.5) and collected in 10 fractions of 1 ml each. Fractions found positive for protein presence at the spectrophotometer were combined and glycerol were add to a final concentration of 10%. Finally, the antibody solution was brought to a neutral pH using 1 M Na_2_HPO_4_ (pH = 9.0) and dialyzed overnight against a buffer containing 20 mM NaH_2_PO_4_, 150 mM NaCl, and 45% glycerol (pH = 7.2). Final antibody concentration was estimated from the measured absorbance at 280 nm.

### Plasmids, Cell Culture, Transfection, and Western Blotting

Cloning of constructs for the mammalian expression of GPRC5A, GPRC5B, GPRC5C, GPRC5D, was previously described (Watkins and Orlandi, 2021). HEK293T/17 cells were used for transfection and western blotting analysis. Cells were cultured at 37°C with 5% CO_2_ in DMEM supplemented with 10% FBS, minimum Eagle’s medium non-essential amino acids, 1 mM sodium pyruvate, and antibiotics (100 units/mL penicillin and 100 μg/mL streptomycin). Two million cells/well were plated in 6-multiwell plates and transfected using polyethyleneimine (PEI, mw 25,000 kDa, Thermo Scientific) and used 24 h later. For western blot analysis, cells were harvested and lysed in ice-cold immunoprecipitation buffer (300 mM NaCl, 50 mM Tris–HCl, pH 7.4, 1% Triton X-100, complete protease inhibitor and phosphatase inhibitor mixture) by sonication. Protein concentration of each sample was quantified using Pierce 660 nm Protein Assay Reagent and equalized to the same concentration. Samples were incubated at 42°C for 10 min and then 10 μL per well were loaded onto 4–20% gradient Mini-PROTEAN TGX Precast Gels (Bio-Rad, Hercules, CA, United States). The same procedure was applied to analyze brain punches and tissue lysate samples. Mouse brains were dissected in an acrylic rodent brain matrix for coronal sections (Stoelting, #51380). Each single 2 mm coronal slice from each brain region of interest was punched using a sterile biopsy punch with plunger (INTEGRA). Both left and right symmetrical 2 mm punches were retrieved from nucleus accumbens (ACB), caudate putamen (CP), hippocampus (HP), hypothalamus (HY), thalamus (TH), amygdala (AM), and cerebellum (CB). For medial prefrontal cortex (mPFC), a single 3 mm punch was retrieved to obtain equal amount of protein for following immunoblotting analysis. Brain punches were flash frozen in liquid nitrogen and stored at −80°C until use. Tissue samples were isolated from whole brain, lung, liver, kidney, heart, and testis of C57BL/6J mice. 1 mL of IP buffer (300 mM NaCl, 50 mM Tris–HCl, pH 7.4, 1% Triton X-100, complete protease inhibitor and phosphatase inhibitor mixture) was used to homogenize 80 mg of tissue of interest. Our rabbit polyclonal anti-GPRC5B antibodies were used at a 1:5,000 dilution; rat anti-HA 1:5,000 (Sigma-Aldrich, St. Louis, MO, United States, #11867423001); mouse anti GAPDH 1:30,000 (Sigma-Aldrich, St. Louis, MO, United States, #MAB374); mouse anti-PSD95 1:20,000 (Invitrogen, #MA1-046); rabbit anti-Calreticulin 1:5,000 (ABclonal, Woburn, MA, United States, #A1066); rabbit anti-Synaptophysin 1:20,000 (ABclonal, Woburn, MA, United States, #A6344); rabbit anti-mGluR5 1:5,000 (MilliporeSigma, St. Louis, MA, United States, #AB5675); rabbit anti-GluN2A 1:2,000 (Santa Cruz, #sc-1468). Anti-rabbit-HRP antibodies were used in 1:1,000 dilution (Kindle Biosciences, LLC, Greenwich, CT, United States, #R1006); anti-mouse-HRP antibodies were used in 1:1,000 dilution (Kindle Biosciences, LLC, #R1005); anti-rat-HRP antibodies were used in 1:10,000 dilution (Fisher Scientific, Waltham, MA, United States, #112-035-175).

### RNAscope *in situ* Hybridization and Imaging

Mice were sacrificed by cervical dislocation followed by decapitation; brains were quickly isolated, embedded in a mold with Tissue Tek O.C.T. compound (Sakura, #4583), and rapidly frozen in a Petri dish floating on liquid nitrogen. 16 μm coronal sections or 20 μm sagittal sections were obtained by cryosectioning of fresh frozen brains and mounted onto Superfrost Plus microscope slides (Thermo Fisher Scientific). *In situ* hybridization was conducted using RNAscope multiplex fluorescent reagent kit (Advanced Cell Diagnostics, #323133) allowing to detect target mRNA at the single cell level. Probes for mouse *Gprc5b* (Mm-Gprc5b-C1 #317461), mouse *Slc17a7* (Mm-Slc17a7-C2 #416631-C2), *Gad1* (Mm-Gad1-C3 #400951), and *Chat* (Mm-Chat-C2 #408731) were designed and purchased from the manufacturer and the experimental procedure was performed following manufacturer’s instructions. Probes were assigned to fluorophores Alexa 488, Atto 550, and Atto 647. For lower magnification imaging of *in situ* hybridization of coronal and sagittal brain sections serving as overview, we used RNAscope multiplex fluorescent reagent kit v2 (Advanced Cell Diagnostics, #323100) with Opal 690 fluorophore (Akoya Biosciences, Marlborough, MA, United States, FP1497001KT) assigned to Mm-Gprc5b-C1 probe for signal amplification. DAPI was used for nucleus counterstaining. Control probes were used for confirmation of sample mRNA preservation and non-specific labeling. As per manufacturer’s instructions, the following three positive control probes were used: Polr2a (Mm-Polr2a-C1 #312471), PPIB (Mm-PPIB-C2 #313911), and Ubc (Mm-Ubc-C3 #310711) (Supplementary Figure 1A). Negative control probe against DapB gene (#310043) from the *Bacillus subtilis* strain SMY was used to evaluate non-specific hybridization (Supplementary Figure 1B). Images were obtained by all-in-one fluorescence microscope Keyence X-800E using a 10× objective for stitched images serving as overview and a 40× objective for images used for expression quantification and statistical analysis. All images used for direct comparisons were acquired with the same intensities of illumination for corresponding channels.

### *Gprc5b* mRNA Expression Analysis

For each brain region of interest, four sections per animal and 2–4 images per section were taken for the following four fluorophores: DAPI, Alexa 488, Atto 550, and Atto 647. Nuclei in brain sections were identified by DAPI staining and used to create a mask twice the area of DAPI with Fiji software (SciJava Consortium). Staining with *Slc17a7*, *Gad1*, and *Chat* were used to identify glutamatergic, GABAergic, and cholinergic neurons, respectively. *Gprc5b* mRNA expression was calculated as ratio of dot signals within each cell to the area of the identified mask. Using this method, expression of *Gprc5b* mRNA in glutamatergic neurons, GABAergic neurons, and striatal cholinergic interneurons was analyzed. For neuronal population distribution analysis, we developed a MATLAB script, determining relative frequency of the neuronal population of interest that express certain amount of *Gprc5b* mRNA. To compare expression levels of *Gprc5b* mRNA within neuronal populations in certain brain regions, we conducted Wilcoxon-Rank Sum two-sided test using the abovementioned MATLAB script.

### Biochemical Brain Fractionation

Cellular fractionation was performed from adult wild type C57BL/6J mouse brain as previously described (Gardoni et al., 1998; Smalla et al., 2013). All procedures were conducted at 4°C in presence of phosphatase and protease inhibitors (Roche). Brain homogenate (Homogenate) was obtained lysing mouse brains in 10 mL/g of lysis buffer containing 0.32 M sucrose and HEPES 5 mM pH 7.4. The lysate was then spun for 10 min at 1,000 g. The supernatant (S1) was collected aside, while the pellet (P1) was resuspended in 10 mL/g of lysis buffer. The lysate was centrifuged again for 10 min at 1,000 × *g* to obtain a supernatant S1′ and pellet P1′. The supernatant S1 was combined with S1′ and centrifuged at 12,000 × *g* for 20 min to obtain a supernatant S2 and pellet P2. The pellet P2 was resuspended and spun at 12,000 g for 20 min to obtain supernatant S2′ and pellet P2′. Supernatants S2 and S2′ were combined and spun at 100,000 *g* for 1 h to obtain a supernatant (cytosolic fraction) and a pellet (Endoplasmic Reticulum-Golgi fraction, ER-Golgi). The pellet P2′ (crude membrane fraction) was resuspended in 1.5 mL/g of a buffer containing 0.32 M sucrose, 5 mM Tris–HCl pH 8.1 and then disposed on 0.85 M/1 M/1.2 M sucrose gradients and spun at 85,000 g for 2 h. The following fractions were collected: between 0.32 and 0.85 M myelin (Myelin); 0.85 M/1 M the light membranes enriched in Endoplasmic Reticulum, Golgi components and other uncharacterized membranes (Light Membranes) (Smalla et al., 2013); the fraction between 1.0 and 1.2 M contained synaptosomes (SYN). Synaptosomes were diluted with an equal volume of 1% Triton X-100 in 0.32 M sucrose containing 1 mM HEPES and stirred for 15 min. The resuspension was spun at 82,500 g for 45 min. The pellet (Triton insoluble post-synaptic fraction, TIF) was resuspended and overlaid on a sucrose gradient (1.0 M/1.5 M/2.1 M) and centrifuged at 100,000 × *g* at 4°C for 2 h. The fraction between 1.5 and 2.1 M (PSD1) was removed and diluted with an equal volume of 1% Triton X-100 and 300 mM KCl. PSD2 was finally collected by centrifugation at 100,000 × *g* at 4°C for 45 min and stored at −80°C until processing. Protein content of the samples was quantified using Pierce 660 nm Protein Assay Reagent. After measuring protein concentration, all samples were standardized at 1 μg/μl concentration and the same protein amount was loaded in each lane. Three independent replicates were performed.

### Deglycosylation Assay

Cellular and tissue samples were quantified for protein concentration using Pierce 660 nm Protein Assay Reagent and the same protein amount was subjected to deglycosylation through the NEB Protein Deglycosylation Mix II (#P6044) following the manufacturer’s non-denaturing protocol. Finally, same protein amounts were loaded in each lane for SDS-PAGE.

### Statistical Analysis

GraphPad Prism software (Version 8) was used to generate graphs of percentage of *Gprc5b* positive cells, MATLAB (R2021a Academic Use) scripts for generating distribution histogram and performing Wilcoxon-Rank Sum two-sided test. Scripts are available upon request. Data were reported in the format of mean ± SEM.

## Results

### GPRC5B Is Enriched in Mouse Brain

**FIGURE 2:**
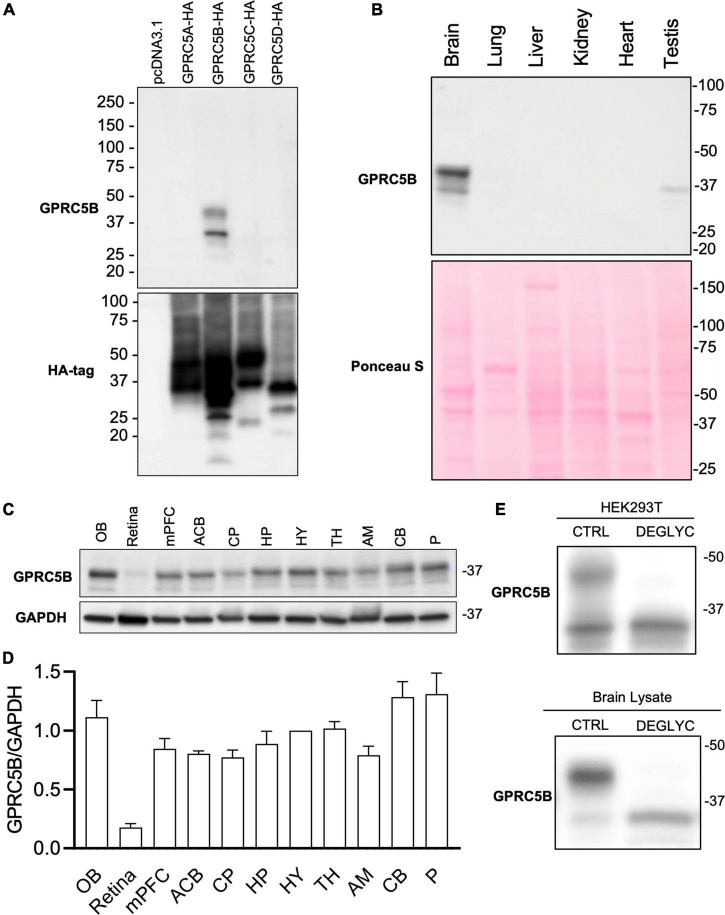
GPRC5B is enriched in the brain. **(A)** GPRC5B antibodies are highly specific. Western blot analysis of HEK293T cells transfected with constructs expressing HA-tagged GPRC5A, GPRC5B, GPRC5C, and GPRC5D along with empty vector pcDNA3.1 using specific anti-HA antibodies show expression of all the constructs (bottom panel). Western blot using our polyclonal anti-GPRC5B antibodies shows specific signal only in GPRC5B-transfected cell lysates. **(B)** Western blot analysis of GPRC5B expression in tissue lysate from brain, lung, liver, kidney, heart, and testis. Ponceau S staining was used as a loading control. **(C)** Brain lysates from different regions were tested for expression of GPRC5B. **(D)** Quantification of GPRC5B expression levels within different brain regions normalized to GAPDH. **(E)** Deglycosylation assay on transfected cells (top panel) and whole brain lysate (bottom panel) followed by immunoblot of GPRC5B. Glycosylated forms located above 37 kDa from both transfected cells and brain tissue lysates collapse to the unmodified forms after the treatment. OB, olfactory bulb; mPFC, medial prefrontal cortex; ACB, nucleus accumbens; CP, caudate putamen; HP, hippocampus; HY, hypothalamus; TH, thalamus; AM, amygdala; CB, cerebellum; P, Pons.

Given a lack of specificity or ineffectiveness of available commercial antibodies to recognize mouse GPRC5B we generated new rabbit polyclonal antibodies. To test the specificity of our affinity-purified GPRC5B polyclonal antiserum, we conducted western blotting against lysates of HEK293 cell lines transfected with GPRC5A-HA, GPRC5B-HA, GPRC5C-HA, and GPRC5D-HA, along with the empty vector pcDNA3.1 (Figure 2A). We detected two bands with molecular weights ranging between 25 and 50 kDa only in lysates from GPRC5B-HA transfected cells, while no bands were visible in lysates from cells expressing the other constructs (Figure 2A, top panel). The predicted molecular weight of the transfected GPRC5B-HA after signal peptide cleavage corresponds to 42.8 kDa and is compatible with the observed bands at approximately 30 and 42 kDa (Stothard, 2000). To further confirm that the other members of the GPRC5 group were properly expressed in transfected cells we performed western blotting using anti-HA antibodies on the same lysates (Figure 2A, bottom panel).

We next investigated the tissue distribution of GPRC5B protein in brain, liver, lung, heart, kidney, and testis using our validated anti-GPRC5B antibodies (Figure 2B, top panel). We found a high level of protein expression in brain lysate while nearly undetectable bands were observed in liver, lung, heart, and kidney. Interestingly, a weak expression was detected in testis lysate. Ponceau S staining confirmed an even protein loading across samples (Figure 2B, bottom panel). Our study confirms at the protein level previous reports about GPRC5B mRNA distribution, as well as data from available RNA sequencing databases, showing enrichment in the brain (Bräuner-Osborne and Krogsgaard-Larsen, 2000; Robbins et al., 2000, 2002; Cool et al., 2010).

### GPRC5B Distribution Across Different Brain Regions

We further sought to characterize GPRC5B distribution across the brain with immunoblotting. To this aim, our validated polyclonal GPRC5B antibodies were applied to tissue lysates of punches from different brain regions (Figure 2C). A detailed quantification of GPRC5B expression levels across 11 brain regions was calculated as a normalized ratio to hypothalamus (HY) expression (Figure 2D). We observed that the olfactory bulb (OB), the pons (P), and the cerebellum (CB) abundantly expressed GPRC5B protein, while it was barely detected in retina lysates. A band slightly higher than 37 kDa led us to speculate about potential post-translational modifications, namely, glycosylation. Therefore, we deglycosylated GPRC5B protein samples from both HEK293T transfected cells and brain lysates and we found that the higher band previously appearing on our immunoblots disappeared after treatment (Figure 2E).

**FIGURE 3:**
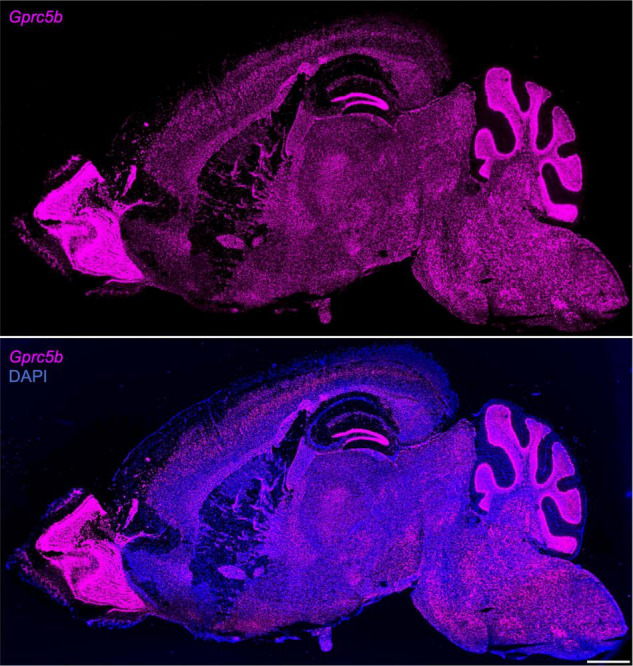
Sagittal overview of *Gprc5b* mRNA distribution throughout mouse brain. Sagittal sections of adult C57BL/6J mouse brain using RNAscope *in situ* hybridization (10×, stitched) hybridized with *Gprc5b* probe **(top panel)** and merged image of the section counterstained with DAPI **(bottom panel)**. Scale bar: 1 mm.

**FIGURE 4:**
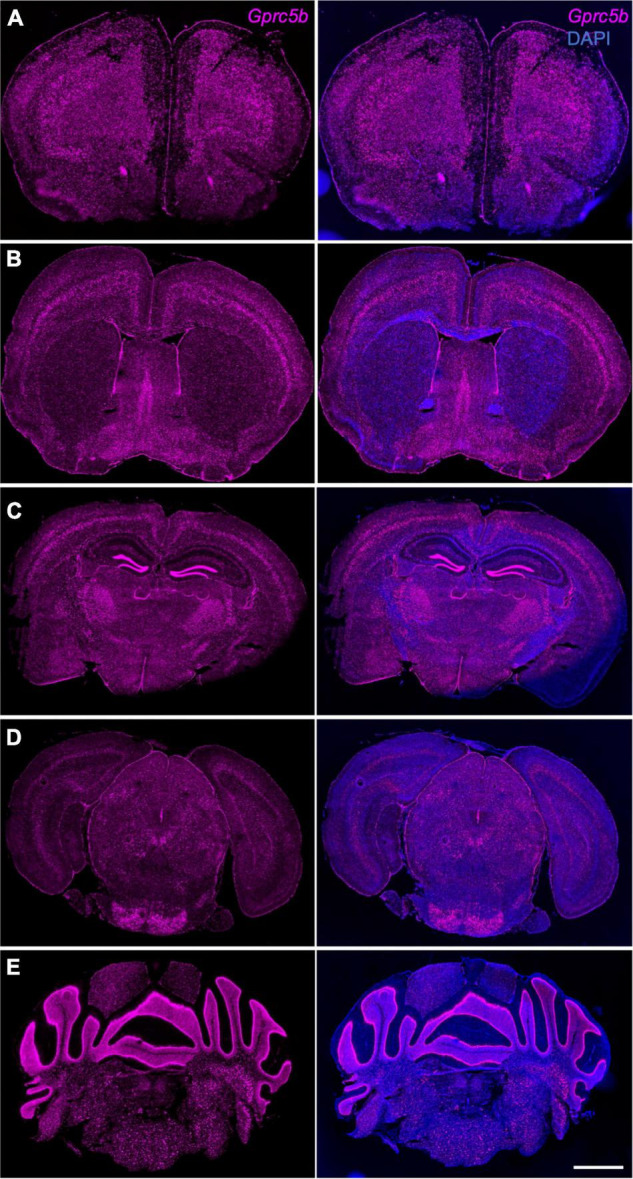
*Gprc5b* mRNA distribution patterns determined by *in situ* hybridization. Coronal mouse brain sections showing *Gprc5b* mRNA expression is highly abundant in layer 5 of the cortex **(A–C)**, sparsely expressed in striatal regions **(B)**, highly expressed within SEZ, SVZ, and SGZ **(A–C)**, and enriched in pontine nuclei as well as in cerebellar Purkinje cells **(D,E)**. Scale bar: 500 μm. SEZ, subependymal zone; SVZ, subventricular zone; SGZ, subgranular zone.

Next, we conducted a comprehensive spatial profiling of *Gprc5b* expression at the mRNA level across different regions of adult mouse brain using RNAscope *in situ* hybridization assay. Overall, *Gprc5b* mRNA signal was widely distributed throughout the mouse brain, while particularly intense in olfactory bulb, hippocampal dentate gyrus (DG), cerebellum, and pons (Figure 3). In addition, coronal mouse brain sections showed that *Gprc5b* mRNA was enriched in cortical layer 5, with only moderate expression in layer 2/3 and layer 6, whereas very low expression was detected in layer 1 (Figures 4A–C). *Gprc5b* mRNA expression was also prominent in brain regions associated with adult neurogenesis including the ependymal cell layers in the ventricular-subventricular zone (V-SVZ) as well as subgranular zone (SGZ), and subependymal zone (SEZ) (Figures 4A–C). In the striatum, only low to moderate expression was detected in both CP and in nucleus ACB (Figure 4B). Finally, staining for *Gprc5b* mRNA was very dense in the pontine nuclei (Figure 4D) as well as in the cerebellar Purkinje layers (Figure 4E). Our analysis of *Gprc5b* distribution pattern across brain regions appears to be largely overlapping to experimental data reported in the Allen Brain Atlas data portal (Supplementary Figure 2).

**FIGURE 5:**
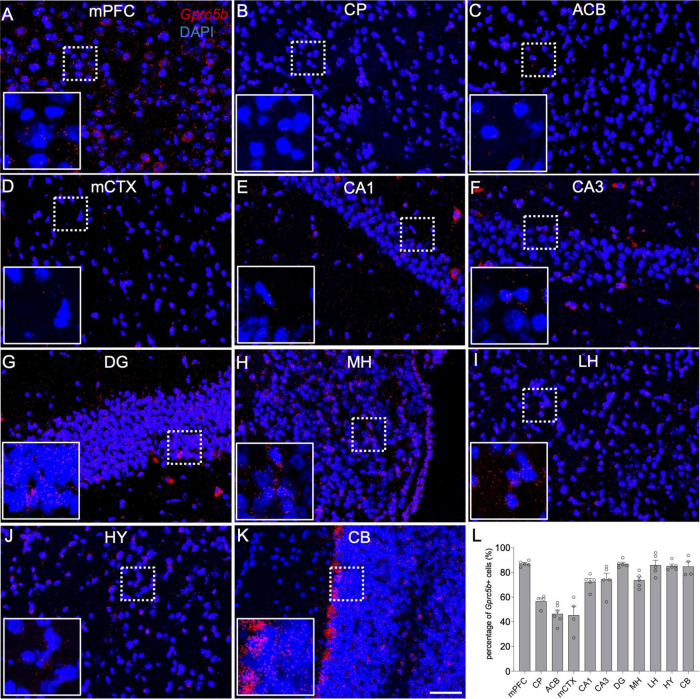
Representative images demonstrating spatial profiling of *Gprc5b* expression on coronal sections from adult mice. **(A)** Medial prefrontal cortex (mPFC), **(B)** caudate putamen (CP), **(C)** nucleus accumbens (ACB), **(D)** motor cortex (mCTX), **(E)** hippocampal CA1, **(F)** hippocampal CA3, **(G)** hippocampal dentate gyrus (DG), **(H)** medial habenula (MH), **(I)** lateral habenula (LH), **(J)** hypothalamus (HY), **(K)** cerebellum (CB). **(L)** Percentage of *Gprc5b* positive cells across the abovementioned regions. Each data point represents calculated percentage of *Gprc5b* positive cells from a single section sampled from 2 to 4 animals and results are reported as mean ± SEM. Scale bar: 50 μm.

The abovementioned dense signals may result from a small number of cells expressing high levels of *Gprc5b* mRNA or from the presence of many cells exhibiting a moderate amount of *Gprc5b* mRNA. To address this question, we carried out a detailed quantification of the percentage of *Gprc5b* positive cells across selected relevant brain regions (Figures 5A–K). More than 80% of the cells showed positive expression of *Gprc5b* in medial prefrontal cortex (mPFC; 86.92%), DG (86.86%), lateral habenula (LH; 85.94%), hypothalamus (HY; 85.10%), and CB (84.80%) (Figure 5L). *Gprc5b* positive cells in the hippocampal regions CA1 and CA3 and in the medial habenula (MH) were, respectively, 72.22, 74.24, and 73.58% (Figure 5L). In contrast, the percentage of *Gprc5b* positive cells in CP, ACB, and motor cortex (mCTX) was, respectively, 56.63, 46.45, and 45.40% (Figure 5L).

### GPRC5B Distribution Within Neuronal Populations

Investigations about GPRC5B distribution across different brain regions provide valuable information, however, further cell-type specific characterization could direct future functional studies more precisely. Thus, we dissected the pattern of *Gprc5b* distribution in various neuronal cell types such as excitatory and inhibitory neurons. To this purpose, we conducted RNAscope dual *in situ* hybridization experiments with the corresponding neuronal biomarkers. Here, solute carrier family 17 member 7 (*Slc17a7*) probes were used as marker for glutamatergic excitatory neurons, glutamic acid decarboxylase 1 (*Gad1*) probes for GABAergic inhibitory neurons, and choline acetyltransferase (*Chat*) probes for striatal cholinergic interneurons.

#### Cortical Regions: Medial Prefrontal Cortex and Motor Cortex

**FIGURE 6:**
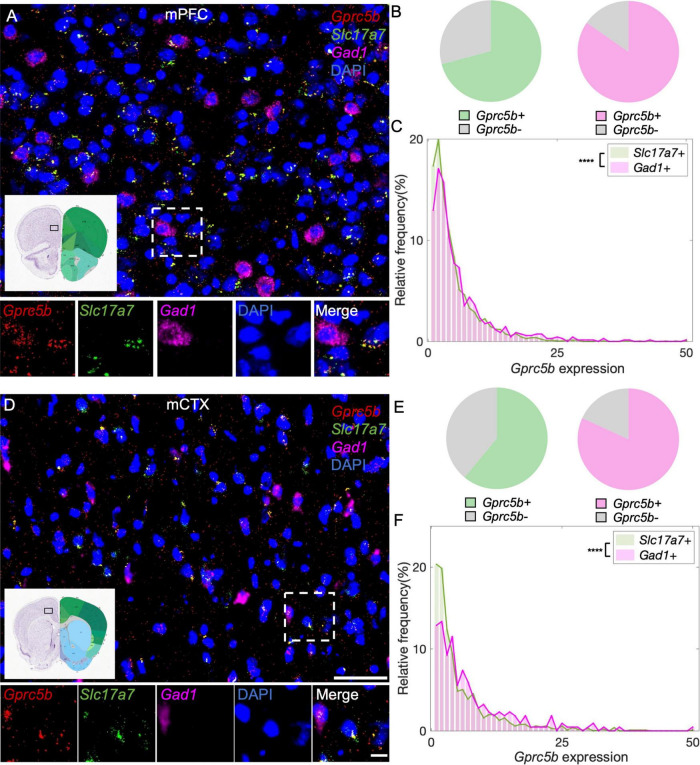
Distribution of *Gprc5b* mRNA within glutamatergic and GABAergic neurons in cortical regions. Representative *in situ* hybridization images indicating *Gprc5b* mRNA distribution in mPFC **(A)** and in mCTX **(D)**. *Gprc5b* was stained in red, *Slc17a7* as a marker for glutamatergic neurons in green, *gad1* as a marker for GABAergic neurons in magenta, and DAPI for nuclei in blue. Pie charts indicating percentage of *Gprc5b* positive neurons in mPFC **(B)** and mCTX **(E)** where *Gprc5b* positive glutamatergic neurons are indicated in green while *Gprc5b* positive GABAergic neurons are in magenta. **(C)** Frequency distribution analysis indicating *Gprc5b* mRNA expression in mPFC within glutamatergic and GABAergic neuronal populations (Bin width = 1, sampled from 2462 glutamatergic neurons and 686 GABAergic neurons of 2–4 animals, *p* < 0.0001 from two-sided Wilcoxon–Rank sum test). **(F)** Frequency distribution analysis of *Gprc5b* mRNA expression in mCTX within glutamatergic and GABAergic neurons (Bin width = 1, sampled from 1013 glutamatergic neurons and 217 GABAergic neurons of 2–4 animals, *p* < 0.0001 from two-sided Wilcoxon–Rank sum test). Scale bar: 50 μm; scale bar of magnified images: 10 μm; reference brain images are from Allen brain atlas. ****p < 0.0001.

The mPFC is a pivotal cortical region whose physiological function encompass regulation of cognition, motivation, and emotions. The two primary neuronal types hosted in the mPFC are glutamatergic pyramidal neurons and GABAergic interneurons (Sun et al., 2019). With our analysis, we found that about 71% of glutamatergic neurons in mPFC express *Gprc5b* and about 85% of GABAergic neurons are positive for *Gprc5b* (Figure 6B). Furthermore, we conducted a detailed frequency distribution analysis of *Gprc5b* expression within these two neuronal populations showing that GABAergic neurons express higher levels of *Gprc5b* than glutamatergic ones as the representative image suggested (Figures 6A,C). mCTX is mainly involved in the planning, controlling, and executing of voluntary body movement. Motor cortical regions are broadly composed of excitatory glutamatergic pyramidal neurons and local inhibitory GABAergic interneurons. Although accounting only for a small fraction of the cortical neurons, GABAergic interneurons play a crucial role in tuning and sculpting the cortical networks (Tremblay et al., 2016). Our analysis showed that approximately 61% of the glutamatergic neurons in the mCTX express *Gprc5b* while around 82% of GABAergic interneurons are positive for *Gprc5b* (Figure 6E). Similar to what observed in the mPFC, GABAergic interneurons express significantly higher levels of *Gprc5b* mRNA than glutamatergic neurons (Figures 6D,F).

#### Hippocampal Regions: Cornu Ammonis 1, Cornu Ammonis 3, and Dentate Gyrus

**FIGURE 7:**
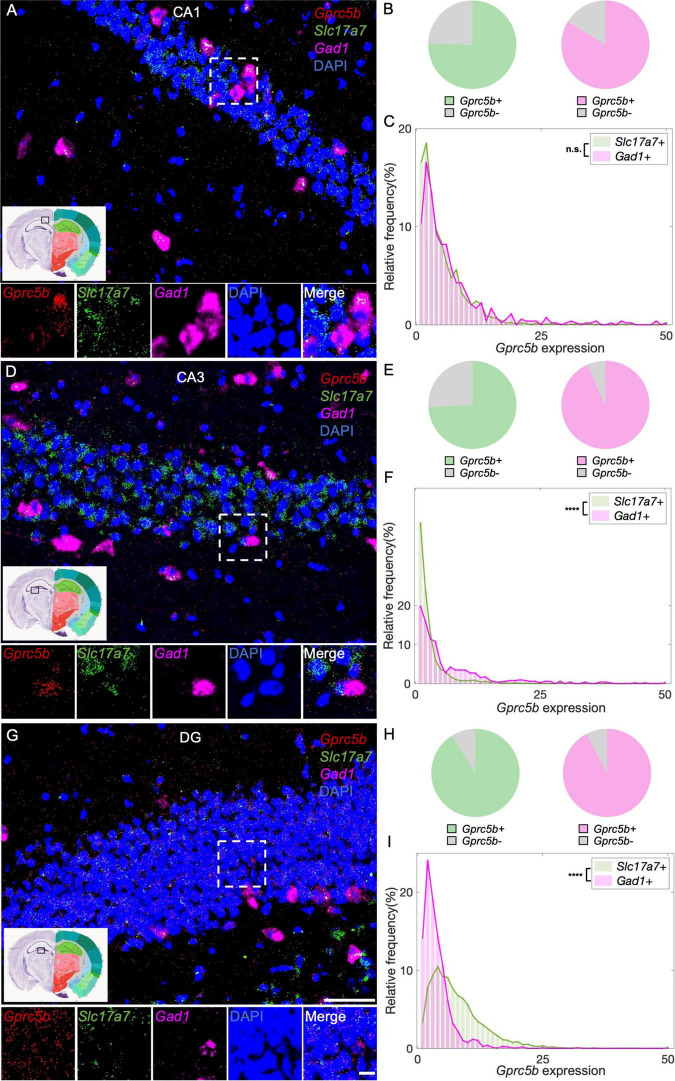
Three hippocampal regions display distinct patterns of distribution of *Gprc5b* mRNA within excitatory and inhibitory neurons. Representative *in situ* hybridization images showing *Gprc5b* mRNA distribution in CA1 **(A)**, CA3 **(D)**, and DG **(G)**. *Gprc5b* was probed in red, glutamatergic neurons marked by *Slc17a7* probe in green, GABAergic neurons indicated by *Gad1* probe in magenta, nuclei by DAPI in blue. Pie charts show fractions of *Gprc5b* positive neurons in each of the glutamatergic or GABAergic neuronal populations, across CA1 **(B)**, CA3 **(E)**, and DG **(H)**. **(C)** Frequency distribution analysis demonstrating *Gprc5b* mRNA expression in CA1 within glutamatergic and GABAergic neuronal populations (Bin width = 1, sampled from 2033 glutamatergic neurons and 538 GABAergic neurons of 2–4 animals, *p* = 0.6486). **(F)** Frequency distribution analysis of *Gprc5b* mRNA expression in CA3 within glutamatergic and GABAergic neurons (Bin width = 1, sampled from 1759 glutamatergic neurons and 520 GABAergic neurons of 2–4 animals, *p* < 0.0001). **(I)** Frequency distribution analysis of *Gprc5b* mRNA expression in DG within glutamatergic and GABAergic neurons (Bin width = 1, sampled from 4640 glutamatergic neurons and 1426 GABAergic neurons of 2–4 animals, *p* < 0.0001). Scale bar: 50 μm; scale bar of magnified images: 10 μm; reference brain images are retrieved from Allen brain atlas. Two-sided Wilcoxon–Rank sum test was conducted to compare expression within neuronal populations. ****p < 0.0001.

Within the hippocampal formation, Cornu Ammonis 1 (CA1) neurons are responsible for the formation, consolidation, and retrieval of long-term memory (Bartsch et al., 2011). The main type of neurons in CA1 are excitatory pyramidal glutamatergic neurons, the function of which is tuned by a small population of GABAergic interneurons (Alkadhi, 2019). According to our analysis of CA1, about 76% of glutamatergic neurons and 84% of GABAergic neurons are positive for *Gprc5b* (Figure 7B) with a comparable distribution of *Gprc5b* expression within the two populations (Figures 7A,C). Compared to other hippocampal formations, the CA3 region is characterized by a richer interconnection in the subfield and neighboring projection to excitatory and inhibitory neurons (Lorente de Nò, 1934). From our analysis, approximately 74% of CA3 glutamatergic neurons and 93% of GABAergic interneurons express *Gprc5b* (Figure 7E). Within the two populations, *Gprc5b* expression in GABAergic neurons was significantly higher than that in glutamatergic neurons (Figures 7D,F). Glutamatergic densely packed granule neurons are the major cell type in the DG, whose functions are supported by a vast network of GABAergic interneurons (Alkadhi, 2019). Our analysis showed that about 91% of both glutamatergic and GABAergic neurons in the DG express *Gprc5b* (Figure 7H). Overall, glutamatergic granule cells express more *Gprc5b* than GABAergic interneurons in the DG (Figures 7G,I).

#### Striatal Regions: Striatum Dorsal Region and Striatum Ventral Region

**FIGURE 8:**
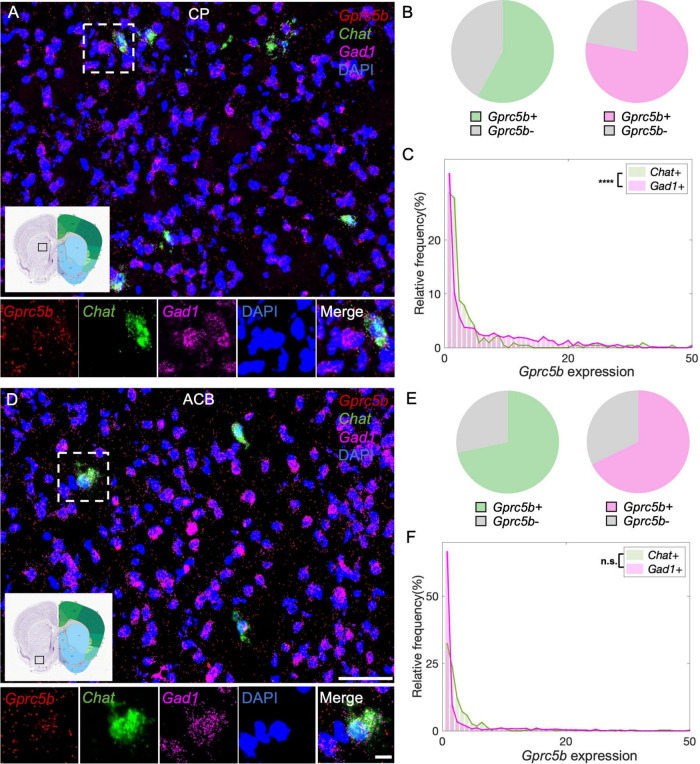
Striatal *Gprc5b* mRNA distribution within cholinergic interneurons and GABAergic medium spiny neurons. Representative *in situ* hybridization images showing *Gprc5b* mRNA distribution in CP **(A)** and ACB **(D)**. *Gprc5b* is in red, cholinergic interneurons marked by *Chat* probe in green, GABAergic MSNs indicated by *Gad1* probe in magenta, nuclei by DAPI in blue. Pie charts showing percentage of *Gprc5b* positive cells within cholinergic (green) and GABAergic (magenta) neuronal populations, across CP **(B)** and ACB **(E)**. **(C)** Frequency distribution graph depicting *Gprc5b* expression within cholinergic and GABAergic neurons in CP (Bin width = 1, sampled from 216 cholinergic interneurons and 1027 GABAergic neurons of 2–4 animals, *p* < 0.0001). **(F)** Frequency distribution graph illustrating *Gprc5b* expression within cholinergic and GABAergic neurons in ACB (Bin width = 1, sampled from 346 cholinergic interneurons and 1603 GABAergic neurons of 2–4 animals, *p* = 0.3592). Scale bar: 50 μm; scale bar of magnified images: 10 μm; reference brain images are retrieved from Allen brain atlas. Two-sided Wilcoxon–Rank sum test was conducted to compare expression within neuronal populations. ****p < 0.0001.

Caudate putamen, or dorsal striatum, is functionally associated with motor systems. Principal neuronal types in CP are GABAergic medium spiny neurons (MSNs), which are classified into D1 or D2 neurons depending on type of dopamine receptors expressed, and that exert opposite effects on movement. Cholinergic interneurons constitute only 1–3% of the whole neuronal population, nonetheless, they play an essential role in regulating the striatal output (Zucca et al., 2018; Mallet et al., 2019). From our analysis, 58% of cholinergic interneurons in the CP express *Gprc5b*, while about 78% of GABAergic MSNs express *Gprc5b* (Figure 8B). Intriguingly, the fraction of cholinergic interneurons positive for *Gprc5b* expression exhibits significantly higher levels of *Gprc5b* than GABAergic MSNs (Figures 8A,C). ACB, or ventral striatum, plays a pivotal role in regulating motivation and reward-related processes (Apicella et al., 1991). Similar to the dorsal striatum, the ACB mainly consists of GABAergic projecting MSNs, along with interspersed cholinergic, fast-spiking and slow-spiking interneurons (Hjorth et al., 2020). Based on our analysis, about 72% of cholinergic interneurons and 68% of GABAergic MSNs are positive for *Gprc5b* (Figure 8E). Consistently, cholinergic interneurons exhibit higher expression levels of *Gprc5b* than GABAergic MSNs (Figures 8D,F).

#### Hypothalamus and Cerebellum

**FIGURE 9:**
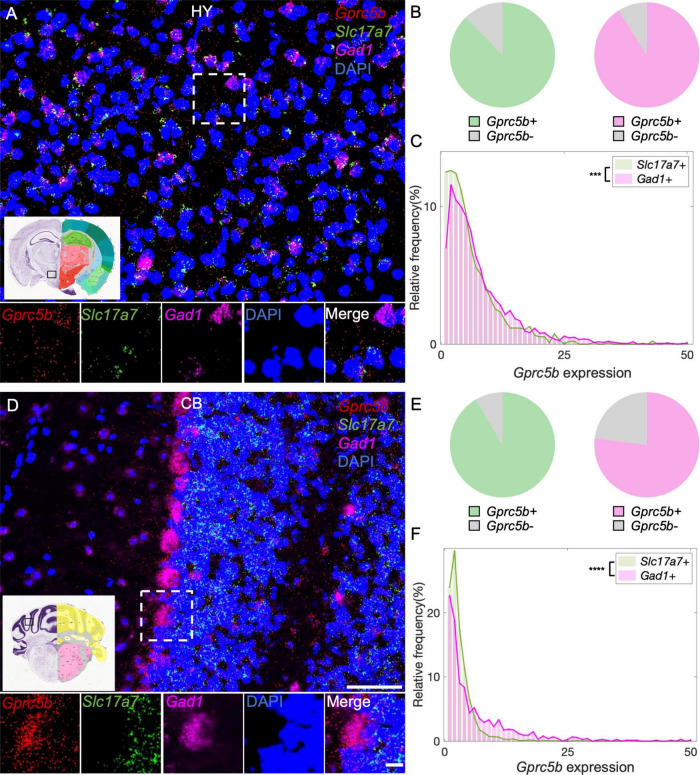
Distribution of *Gprc5b* transcripts in glutamatergic and GABAergic neurons across HY and CB. Representative fluorescent images showing *Gprc5b* mRNA distribution in HY **(A)** and in CB **(D)**. *Gprc5b* was indicated in red, *Slc17a7* as a marker for glutamatergic neurons in green, *gad1* as a marker for GABAergic neurons in magenta, and DAPI for nuclei in blue. Pie charts demonstrating percentage of *Gprc5b* positive neurons in HY **(B)** and CB **(E)** where *Gprc5b* positive glutamatergic neurons are in green while *Gprc5b* positive GABAergic neurons are in magenta. **(C)** Frequency distribution analysis indicating *Gprc5b* mRNA expression in HY within glutamatergic and GABAergic neurons (Bin width = 1, sampled from 943 glutamatergic neurons and 2428 GABAergic neurons of 2–4 animals, *p* < 0.001 from two-sided Wilcoxon–Rank sum test). **(F)** Frequency distribution analysis of *Gprc5b* mRNA expression in CB in glutamatergic and GABAergic neuronal populations (Bin width = 1, sampled from 2888 glutamatergic neurons and 755 GABAergic neurons from 2 to 4 animals, *p* < 0.0001 from two-sided Wilcoxon–Rank sum test). Scale bar: 50 μm; scale bar of magnified images: 10 μm; reference brain images were obtained from Allen brain atlas. ***p < 0.001; ****p < 0.0001.

Hypothalamus plays a critical role in modulating the neuroendocrine system and thus it is essential for the internal homeostasis of body temperature, blood pressure, water intake, and control over the circadian rhythm (Cooper, 1966; Geraldes et al., 2018). Previous single-cell RNA sequencing investigations suggested that principal hypothalamic neuronal types include both glutamatergic and GABAergic neurons and a small number of histaminergic neurons related to modulating circadian rhythm (Chen et al., 2017). Our analysis showed that about 88% of glutamatergic neurons and about 91% of GABAergic hypothalamic neurons express *Gprc5b* (Figure 9B). Once again, GABAergic neurons in this region exhibited higher levels of *Gprc5b* than glutamatergic neurons (Figures 9A,C). CB is responsible for coordination of fluid movement, sequence learning, and cognition (Koziol et al., 2014). GABA-releasing Purkinje cells represent the unique projecting neurons in the cerebellar cortex. From our analysis, about 92% of the glutamatergic neurons and 77% of the GABAergic neurons in the CB express *Gprc5b* with signal showing high enrichment in the Purkinje cells (Figures 9D,E). Overall, the GABAergic neurons in the CB exhibited higher levels of *Gprc5b* than the glutamatergic population (Figures 9D,F).

### GPRC5B Subcellular Localization in Mouse Brain

**FIGURE 10:**
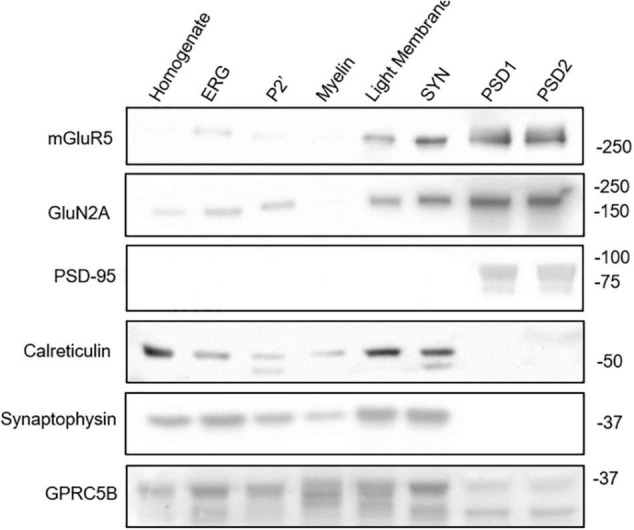
GPRC5B subcellular localization. Biochemical brain fractionation followed by western blot analysis were conducted to investigate the subcellular localization of GPRC5B. PSD95 immunoblot was used to confirm post-synaptic density, calreticulin for ER-Golgi, and synaptophysin for pre-synaptic membranes. Western blots of the synaptic GPCR mGluR5 and of the NMDAR subunit NR2A served as a reference for subcellular distribution. Detailed explanation of distinct fractions is provided in the Section “Materials and Methods.”

A comprehensive mass spectrometry analysis previously identified GPRC5B localization in the PSD (Trinidad et al., 2008), while imaging studies using cells transfected with GPRC5B constructs showed enrichment in intracellular compartments, namely, ER and Golgi, or at the plasma membrane depending on the cell type transfected (Robbins et al., 2000; Bräuner-Osborne et al., 2001; Kim et al., 2012, 2018; Kim and Hirabayashi, 2018; Carvalho et al., 2020). Moreover, another study reported localization of GPRC5B in exosomes after induction with hepatocyte growth factor in a kidney-derived cell line (Kwon et al., 2016). Here, we performed a biochemical fractionation of whole mouse brain to assess subcellular compartments enriched in GPRC5B by western blot. We verified fraction purity through different markers such as PSD-95 for the PSD fractions (PSD1, PSD2), and synaptophysin for the synaptosomal (SYN) fraction (Figure 10 and Supplementary Figure 3). Interestingly, GPRC5B signal indicates localization in the ER-Golgi, light membrane fractions, and also in the PSD fractions (Figure 10 and Supplementary Figure 3). Noteworthy, both ER-Golgi and light membranes contain ER portions (Smalla et al., 2013) as also confirmed by the ER marker calreticulin (Müller-Taubenberger et al., 2001). Overall, the fractionation results support previous information about GPRC5B receptor localization in the ER and confirmed its presence at the PSD.

## Discussion

In the present study, distribution analysis of GPRC5B confirmed its high expression at the protein level in the CNS, especially within OB, CB, and Pons. Furthermore, a detailed *in situ* hybridization analysis also revealed enrichment in smaller areas such as DG, pontine nuclei, cerebellar Purkinje cells, SEZ, SVZ, and SGZ. These observations are in agreement with previous published reports and data from RNA sequencing (Bräuner-Osborne and Krogsgaard-Larsen, 2000; Robbins et al., 2002; Cool et al., 2010; Soni et al., 2013). Our thorough analysis of *Gprc5b* expression in excitatory and inhibitory neuronal populations across many brain areas identified expression levels significantly higher in GABAergic neurons than glutamatergic neurons across six brain regions. Previous studies showed that GPRC5B conditional knockout in Purkinje cells, the main GABAergic population in CB, affected the development of distal axons to specific neurons and regulation of synaptic plasticity underlying motor learning (Sano et al., 2018). Consistently, the present study reported intense *Gprc5b* signal at the transcript level, localized in cerebellar Purkinje cells and in the pontine nuclei, regions both associated with motor learning and motor coordination functions. Intriguingly, contrary to the other brain regions investigated, glutamatergic granule cells of the DG showed significantly higher expression of *Gprc5b* compared to GABAergic neurons. This suggests a potential involvement of GPRC5B in the modulation of spatial memory formation and consolidation *via* signaling in DG granule cells through mossy fiber pathway (De Bruyckere et al., 2018). Moreover, enrichment of *Gprc5b* mRNA in SEZ, SVG, and SGZ could lead to speculations about a potential functional role of GPRC5B in adult neurogenesis. This agrees with developmental studies that revealed the involvement of GPRC5B in neuronal differentiation fate decision steps of cortical neural progenitor cells (Kurabayashi et al., 2013).

Our biochemical fractionation performed to define the subcellular localization of GPRC5B in mouse brain indicated that a large amount of GPRC5B is localized intracellularly in regions including ER and Golgi apparatus, while part of it is also found at PSD sites. GPRC5B presence in the PSD confirms previous proteomics analysis from mouse cortex, midbrain, cerebellum, and hippocampus that identified GPRC5B in the PSD of each of these brain regions (Trinidad et al., 2008). On the other side, the presence of GPRC5B in intracellular membrane compartments has been previously observed in transfected cell lines where the localization of tagged versions of GPRC5B was analyzed by immunocytochemistry (Robbins et al., 2000; Bräuner-Osborne et al., 2001; Kim et al., 2012, 2018; Kim and Hirabayashi, 2018; Carvalho et al., 2020). However, depending on the cell line transfected, GPRC5B was also observed as targeted to the plasma membrane (Robbins et al., 2000; Bräuner-Osborne et al., 2001; Carvalho et al., 2020). Molecular mechanisms responsible for the differential intracellular targeting of GPCR5B are currently under scrutiny. Similarly, intracellular signaling cascades activated by this orphan receptor are starting to be explored. Indeed, emerging studies on GPRC5A, a close GPRC5 family member, recently depicted a potential role in endomembrane signaling that may be a common feature of this class of orphan GPCRs (Di Martino et al., 2020; Liccardo et al., 2022). Therefore, it is possible that GPRC5B may serve different roles according to its specific subcellular location. Different levels of glycosylation observed *in vivo* and in transfected cells may also indicate potential mechanisms underlying its membrane targeting and will require further investigations.

Given the relatively limited set of functional data related to the role of GPRC5B in the CNS, the development of novel tools to investigate this and other oGPCRs will be essential in generating new knowledge for future studies. In this direction, this study presents a comprehensive expression profile of GPRC5B in mouse brain and neuronal populations which improves our knowledge and provides guidance for future functional studies.

## Data Availability Statement

The original contributions presented in this study are included in the article/Supplementary Material, further inquiries can be directed to the corresponding author.

## Ethics Statement

The animal study was reviewed and approved by University Committee on Animal Resources (UCAR) at the University of Rochester.

## Author Contributions

CO conceived and designed the study. WF performed the western blots, carried out RNAscope *in situ* hybridizations, developed the MATLAB scripts, and conducted the statistical analysis. LF performed the deglycosylation assays and biochemical fractionation experiments and analyzed the data. CO and WF wrote the manuscript. All authors contributed to the article and approved the submitted version.

## Conflict of Interest

The authors declare that the research was conducted in the absence of any commercial or financial relationships that could be construed as a potential conflict of interest.

## Publisher’s Note

All claims expressed in this article are solely those of the authors and do not necessarily represent those of their affiliated organizations, or those of the publisher, the editors and the reviewers. Any product that may be evaluated in this article, or claim that may be made by its manufacturer, is not guaranteed or endorsed by the publisher.

## Supplementary Material

Supplementary Figure 1 Positive and negative controls for RNAscope analysis of mRNA distribution patterns. In column **(A)** three mouse specific housekeeping gene *Polr2a*, *UBC*, and *PPIB* were used as positive control probes for technical quality and mRNA preservation check. Ubiquitous signals from *Polr2a* (red), *UBC* (green), and *PPIB* (magenta) were observed in both 10× stitched images and images at higher magnification (insets). In column **(B)** *in situ* hybridization achieved with a probe against the soil bacteria gene *DapB* was used as a negative control for non-specific hybridization in the corresponding three channels. Scale bar: 500 μm.

Supplementary Figure 2 *Gprc5b* mRNA distribution patterns retrieved from Allen brain atlas data portal detected by *in situ* hybridization. **(A)** Sagittal brain section of a C57BL/6J mouse showing *Gprc5b* mRNA distribution. Scale bar: 839 μm. **(B)** Coronal mouse brain sections with comparable anatomical positions to images presented in Figure 4. Scale bar: 1678 μm.

Supplementary Figure 3 Original western blots of mouse brain biochemical fractionation experiments. Three replicates are reported.
