# Supplementary figures and images for: Comprehensive Spatial Profile of the Orphan G Protein Coupled Receptor GPRC5B Expression in Mouse Brain
Source: Front Neurosci. 2022 Jun 23;16:891544. doi: 10.3389/fnins.2022.891544 (PMC9259939; doi:10.3389/fnins.2022.891544)

Suppl. Fig. 1

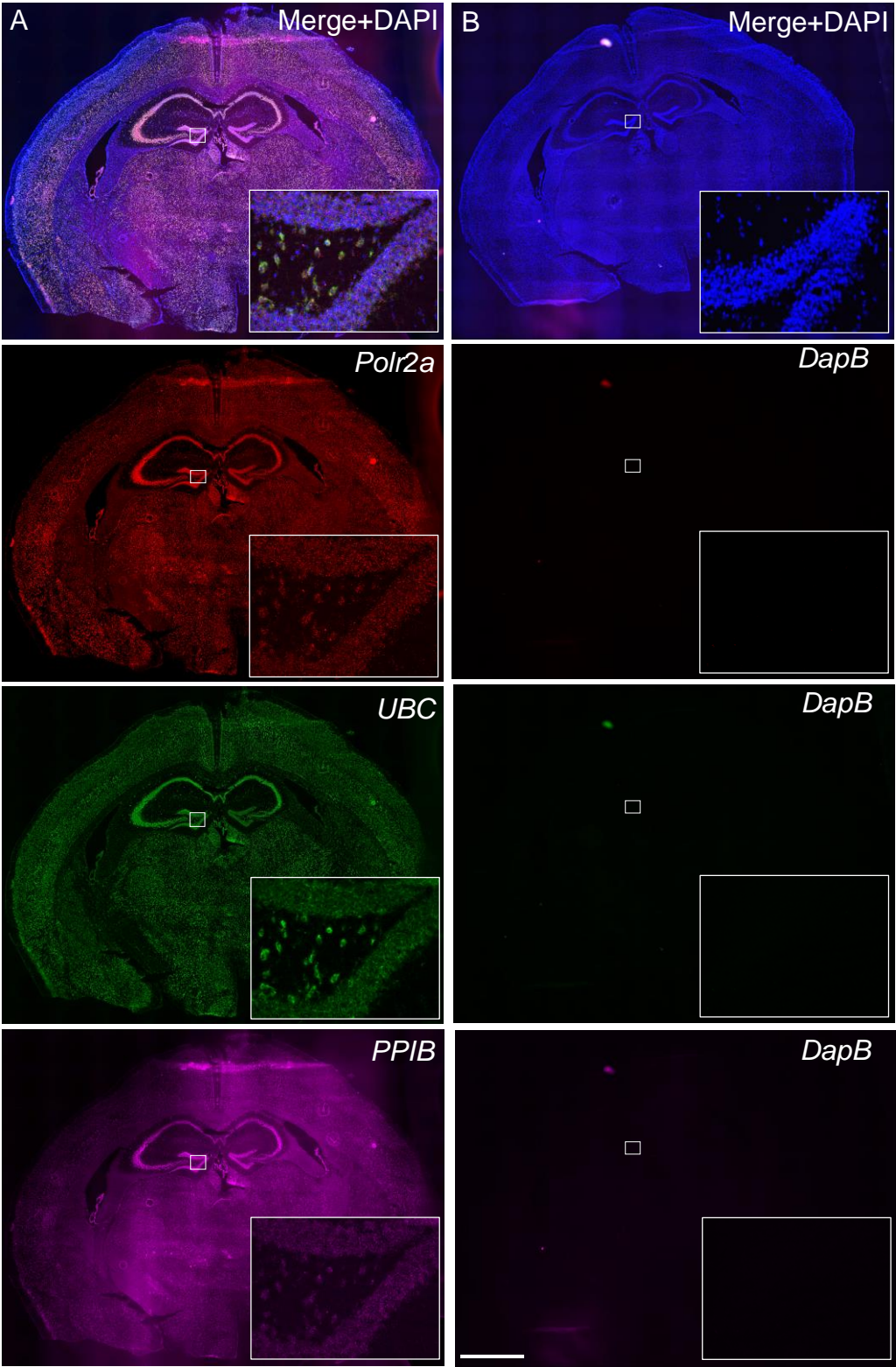

Supplement: Supplementary Figure 1 — Positive and negative controls for RNAscope analysis of mRNA distribution patterns. In column (A) three mouse specific housekeeping gene Polr2a, UBC, and PPIB were used as positive control probes for technical quality and mRNA preservation check. Ubiquitous signals from Polr2a (red), UBC (green), and PPIB (magenta) were observed in both 10× stitched images and images at higher magnification (insets). In column (B) in situ hybridization achieved with a probe against the soil bacteria gene DapB was used as a negative control for non-specific hybridization in the corresponding three channels. Scale bar: 500 μm. [file Image_1.pdf]

Suppl. Fig. 2

A

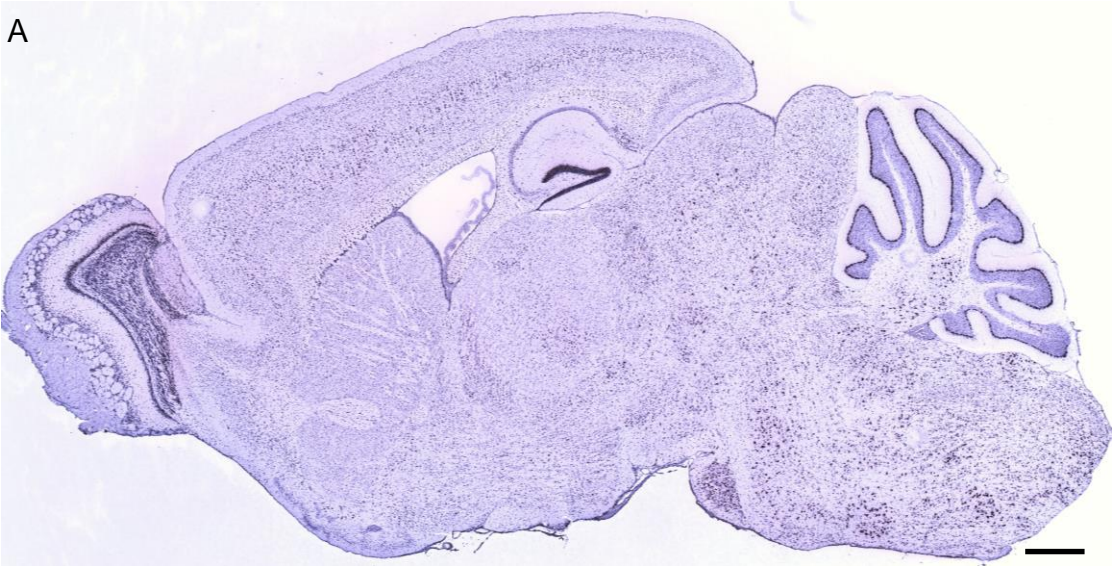

B

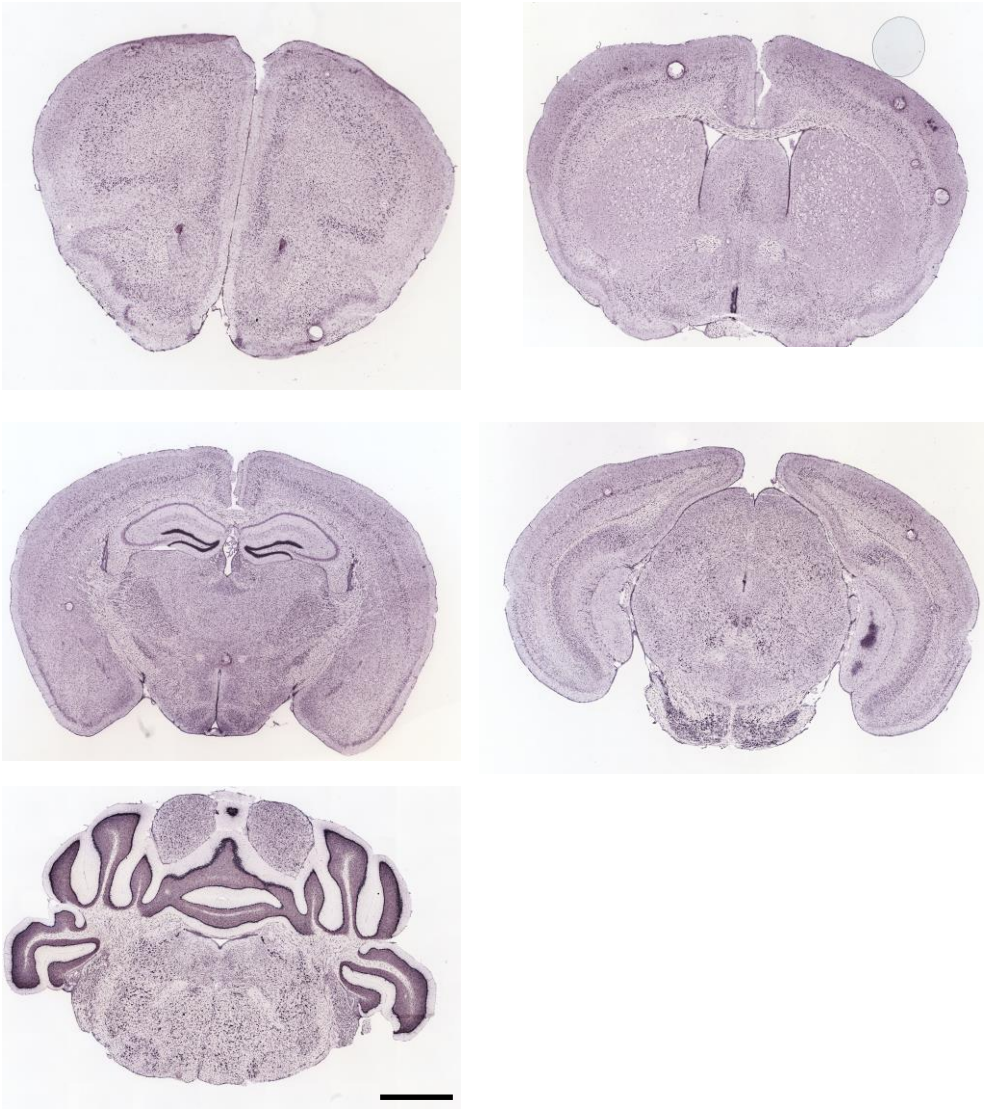

Supplement: Supplementary Figure 2 — Gprc5b mRNA distribution patterns retrieved from Allen brain atlas data portal detected by in situ hybridization. (A) Sagittal brain section of a C57BL/6J mouse showing Gprc5b mRNA distribution. Scale bar: 839 μm. (B) Coronal mouse brain sections with comparable anatomical positions to images presented in Figure 4. Scale bar: 1678 μm. [file Image_2.pdf]

# Suppl. Fig. 3

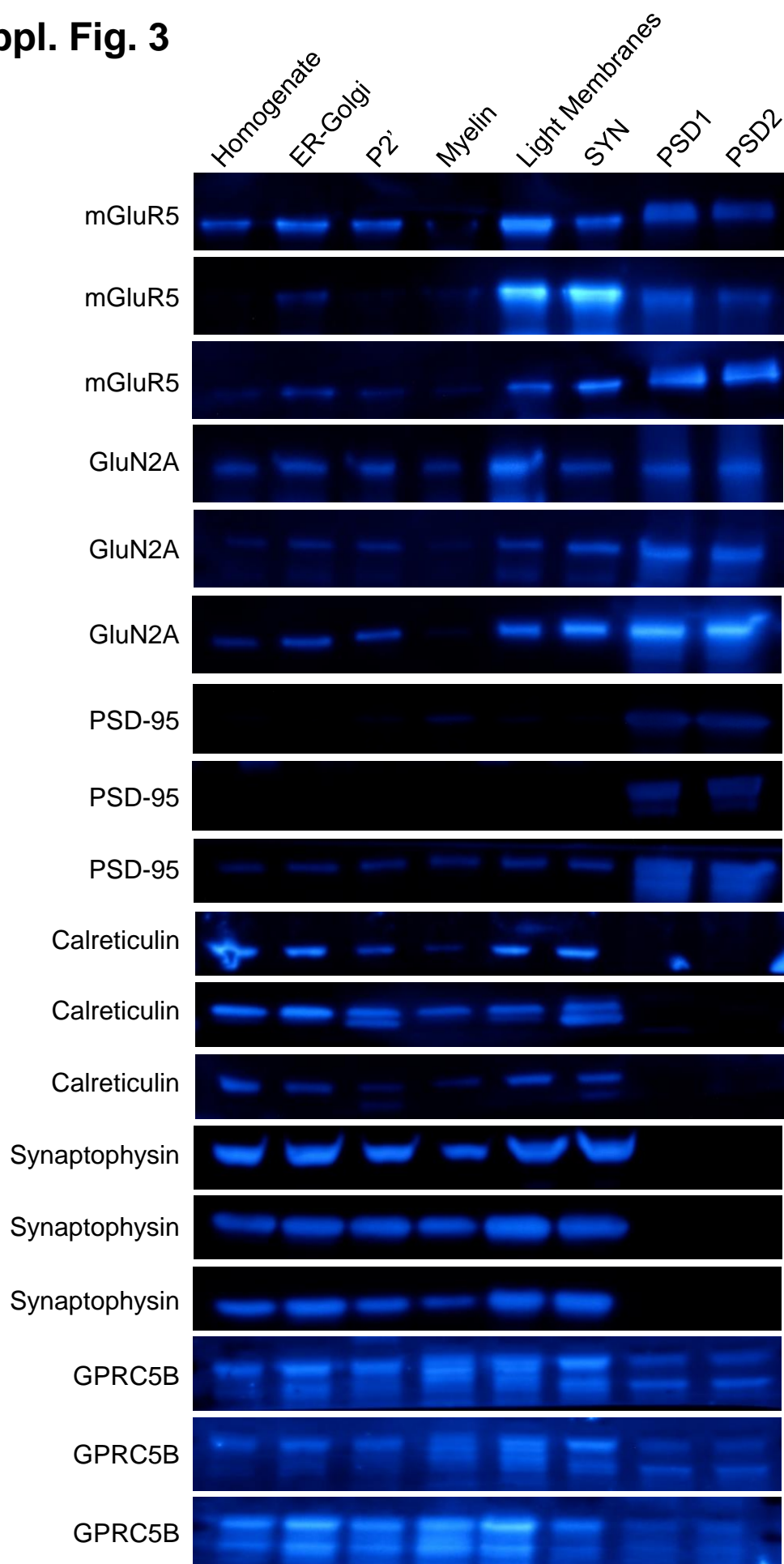

Supplement: Supplementary Figure 3 — Original western blots of mouse brain biochemical fractionation experiments. Three replicates are reported. [file Image_3.pdf]
